# Endothelial Arginine Metabolism in Angiogenesis: Mechanistic Insights from Tissue Repair to Tumor Progression

**DOI:** 10.3390/metabo15110694

**Published:** 2025-10-25

**Authors:** Cristina Arce-Recatala, Roxana Elena Oberkersch

**Affiliations:** 1 Bioengineering Group (iBIO), Valencian International University (VIU), 46002 Valencia, Spain; cristina.arce@professor.universidadviu.com; 2 Department of Oncology, University of Torino, 10060 Candiolo, Italy

**Keywords:** endothelial cells, arginine metabolism, tumor angiogenesis, tissue repair

## Abstract

Angiogenesis, the process of forming new blood vessels from pre-existing vasculature, is essential both during development and in adulthood under physiological and pathological conditions. Therefore, understanding the molecular mechanisms that control angiogenesis has far-reaching implications in developmental biology and the treatment of human diseases. In this context, amino acid metabolism has emerged as a key driver of blood vessel formation. While the role of L-arginine (L-arg) in the cardiovascular system has been extensively described, whether L-arg could serve as a potential metabolite to target during tumor angiogenesis or be exploited to promote tissue regeneration remains unclear. Here, we will describe L-arg metabolism in the vascular context and its crosstalk with angiogenic metabolic pathways. We will also review the main findings regarding the role of L-arg in tissue regeneration and tumor progression, situating L-arg at the center of the discussion on regenerative and preventive vascular medicine.

## 1. Introduction

Angiogenesis is a tightly regulated, multi-step process through which new blood vessels form from existing ones [1,2]. It begins with the so-called angiogenic switch, a critical transition during which dormant or quiescent endothelial cells (ECs) become activated in response to pro-angiogenic signals, triggering their migration and proliferation [3,4]. Angiogenesis is crucial during development as well as in adulthood under pathophysiological conditions. In fact, it is a visible and important component of wound repair [5] and can become dysregulated in various pathological conditions, including atherosclerosis [6], diabetes [7], obesity [8], and tumor progression [9].

During the angiogenic switch, ECs undergo metabolic reprogramming to adapt to their microenvironment and meet their specific functional demands. In fact, some metabolic pathways are crucial to support EC proliferation and migration. For instance, 6-phosphofructo-2-kinase/fructose-2,6-biphosphatase (PFKFB3), a key regulatory enzyme of the glycolytic pathway, controls angiogenesis by elevating glycolysis flux, regulating the cell cycle and protecting ECs from apoptosis [10,11]. But not only is glucose essential for ECs; indeed, fatty acid oxidation (FAO) provides acetyl-CoA, which helps replenish the tricarboxylic acid (TCA) cycle and synthesize nucleotides [12,13]. In recent years, amino acid metabolism has emerged as a key regulator of vascular function and EC behavior. Glutamine, the most abundant nonessential amino acid (NEAA), plays a pivotal role in angiogenesis by serving as a carbon and nitrogen source. It can be metabolized to aspartate, which contributes to pyrimidine biosynthesis, and to asparagine, supporting both nucleotide and protein synthesis required for EC proliferation and migration during vessel formation [14,15]. Also, cysteine, the second most abundant NEAA, has been shown to be critical for EC survival and angiogenic activation. Beyond its role in glutathione synthesis and redox homeostasis, cysteine availability influences the susceptibility of cells to ferroptosis—a regulated form of cell death linked to lipid peroxidation [16]. During angiogenesis, proliferating ECs exhibit increased metabolic demand and oxidative stress, making cysteine-dependent antioxidant pathways essential for maintaining cellular integrity and supporting vessel formation [17].

L-arginine (L-arg) is a semi-essential or conditionally essential amino acid known for its role in protein synthesis and for being a substrate for nitric oxide synthase (NOS) to produce the vascular-protective molecule nitric oxide (NO) [18], and it has well-established functions in the cardiovascular system [19,20,21]. However, its precise functions in sprouting angiogenesis and its connection with key angiogenic metabolic pathways are not yet fully understood. Furthermore, how L-arg metabolism is reprogrammed during processes such as tissue repair and tumor angiogenesis remains largely unexplored. Unraveling these complex metabolic interactions could provide valuable insights into novel therapeutic strategies aimed at modulating angiogenesis in both regenerative medicine and cancer treatment.

Although prior studies have investigated discrete components of endothelial metabolism, the interconnections among these pathways—and their collective influence on angiogenic regulation—have not yet been comprehensively synthesized. To address this gap, the present review consolidates existing knowledge into a unified framework that highlights the interplay between L-arg metabolism and other critical metabolic circuits governing endothelial function.

This review synthesizes the classical roles of L-arg metabolism in the vascular endothelium alongside its metabolic crosstalk with recent advances in tissue regeneration and tumor angiogenesis. It aims to broaden the understanding of L-arg metabolism as a novel and promising driver of vascular biology, particularly in vascular regeneration and tumor angiogenesis, while also raising new questions in the field of amino acid metabolism as potential targets for controlling physiological and pathological angiogenesis.

## 2. Arginine Metabolism and Its Metabolic Crosstalk

L-arg metabolism channels substrates into multiple distinct metabolic pathways, including citrulline regeneration, NO synthesis, polyamine production, the urea cycle, and creatine biosynthesis. This complex regulation is orchestrated by the precise subcellular localization of key enzymes, ensuring coordinated metabolic control.

**Figure 1:**
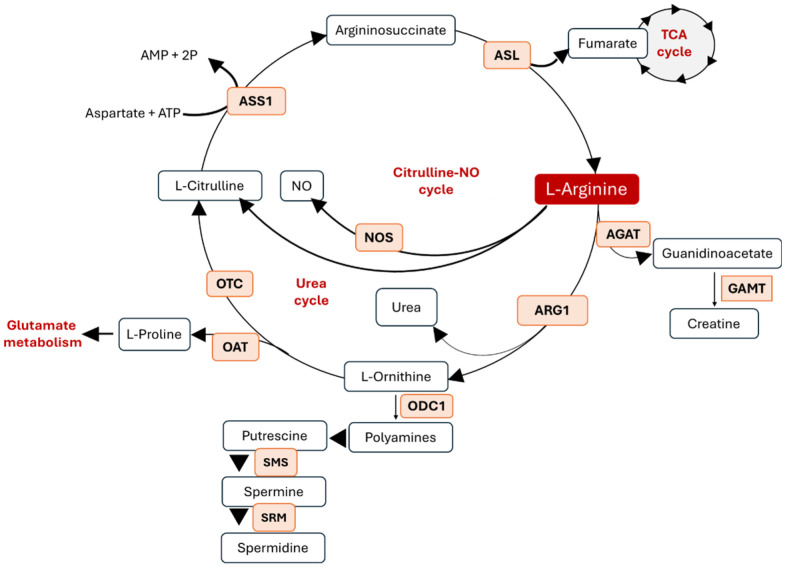
Arginine metabolism and metabolic crosstalk. Abbreviations: AGAT—L-arginine–glycine amidinotransferase, GAMT—guanidinoacetate N-methyltransferase, ARG—arginase, NOS—nitric oxide synthase, ODC1—ornithine decarboxylase 1, ASS1—argininosuccinate synthase 1, OTC—ornithine carbamoyltransferase, SMS—spermine synthase, SRM—spermidine synthase, OAT—ornithine aminotransferase, and ASL—argininosuccinate lyase.

In the cytosol, argininosuccinate synthase 1 (ASS1) and argininosuccinate lyase (ASL) couple citrulline to L-arg regeneration while providing fumarate to the tricarboxylic acid (TCA) cycle. Cytosolic arginase (ARG1) hydrolyzes L-arg to ornithine and urea, linking L-arg metabolism to nitrogen disposal, while its mitochondrial isoform (ARG2) contributes to tissue-specific regulatory functions. NOS, localized in the cytosol, converts L-arg into NO, integrating L-arg flux with vascular signaling and immune function. In mitochondria, arginine–glycine amidinotransferase (AGAT) generates guanidinoacetate, which is further processed by cytosolic guanidinoacetate N-methyltransferase (GAMT) into creatine, connecting L-arg to energy buffering in muscle and brain. Ornithine metabolism also branches into polyamine biosynthesis through cytosolic ornithine decarboxylase (ODC1), followed by spermidine synthase (SRM) and spermine synthase (SMS), which produce polyamines essential for cell growth and nucleic acid stability. Meanwhile, mitochondrial ornithine aminotransferase (OAT) redirects ornithine toward proline and glutamate metabolism, and ornithine transcarbamylase (OTC) integrates L-arg metabolism with the mitochondrial urea cycle [22]. Altogether, the compartmentalized distribution of these enzymes underlies the versatility of L-arg metabolism, positioning it as a central amino acid that connects nitrogen balance, energy homeostasis, signaling pathways, and biosynthetic networks (Figure 1).

A major pathway of L-arg metabolism involves its conversion to NO and citrulline by the NOS enzyme. This reaction occurs in nearly all mammalian cells, such as ECs, macrophages, neurons, muscle cells, adipocytes, enterocytes, and renal epithelial cells. NO acts as a vasodilator, anti-inflammatory, and anti-thrombotic agent, inhibiting leukocyte adhesion, platelet aggregation, and smooth muscle proliferation. Despite endothelial nitric oxide synthase (eNOS) having a low Km for L-arg (2.9 µM), intracellular L-arg concentrations are significantly higher (500–3000 µM) in physiological conditions. This would imply that eNOS could be saturated and that NO production should not be sensitive to changes in extracellular L-arg levels. However, the phenomenon known as the “arginine paradox” reveals that NO production increases despite saturating intracellular L-arg levels; this highlights the complexity of its regulation, involving factors like asymmetric dimethylarginine (ADMA)-mediated NOS inhibition and L-arg-induced tetrahydrobiopterin (BH4) synthesis, a vital NOS cofactor [23,24]. In addition, L-arg is primarily metabolized by NOS to produce NO in M1 macrophages to promote pathogen killing and inflammation. In contrast, M2 macrophages, which are alternatively activated and involved in anti-inflammatory processes and tissue repair, metabolize L-arg via arginase [25,26].

L-arg is also metabolized by arginase to ornithine, which is further converted into polyamines such as putrescine, spermidine, and spermine [27]. These molecules are essential for cell proliferation, gene expression, and tissue remodeling, but their levels must be tightly regulated. The abundance of polyamines in cells is maintained within a narrow range as they have adverse effects if present either in insufficient amounts or in excessive amounts. Insufficient amounts of polyamines inhibit the proliferation and migration of cells, whereas an over-abundance of polyamines results in apoptosis and cell transformation due to their catabolism to toxic reactive aldehydes and reactive oxygen species that enhance oxidative stress on cells [28].

In a physiological context, L-arg also contributes to nitrogen disposal via the urea cycle and supports anabolic processes, such as protein synthesis and creatine synthesis. Emerging evidence shows that L-arg availability influences disease progression. For example, disorders of creatine synthesis, such as those caused by AGAT deficiency, can result in intellectual disability and muscle weakness [29]. ARG1 deficiency is a rare urea cycle disorder that results in persistent hyperargininaemia and a distinct, progressive neurologic phenotype involving developmental delay, intellectual disability, and spasticity, predominantly affecting the lower limbs and leading to mobility impairment [30]. Silencing ASS1 in cancer cells has been shown to suppress the urea cycle and redirect available aspartate into pyrimidine biosynthesis [31].

Beyond the urea cycle, L-arg plays a key role in activating the mechanistic target of rapamycin complex 1 (mTORC1) pathway, which regulates protein synthesis and cell proliferation [32,33]. L-arg is sensed by CASTOR1, a cytosolic L-arg sensor that interacts with GATOR2 to form the CASTOR1-GATOR2 complex. In arginine-rich conditions, the CASTOR1-GATOR2 complex dissociates, allowing the formation of the CASTOR1-arginine complex, which is essential for L-arg-mediated mTORC1 activation. However, in the absence of L-arg, CASTOR1 suppresses mTORC1 activity by interacting with the GATOR2 complex. Upon binding to L-arg, CASTOR1 undergoes a conformational change, leading to the dissociation from GATOR2 and subsequent activation of mTORC1 [34].

Arginine also plays a significant role in glucose metabolism. For instance, L-arg can be metabolized by either ARG or NOS, each leading to different physiological outcomes. Through the arginase pathway, L-arg is converted into urea and ornithine. Urea accumulation has been implicated in pancreatic β-cell dysfunction, contributing to insulin resistance and glucose intolerance. Ornithine, on the other hand, is further metabolized into polyamines and proline via ornithine decarboxylase (ODC) and ornithine aminotransferase (OAT), which may promote a pro-inflammatory state that negatively affects glucose regulation. In contrast, when metabolized by NOS, L-arg produces citrulline and NO. This gas is a key regulator of endothelial function, and it plays an important role in stimulating insulin secretion and enhancing insulin sensitivity. In addition, it is reported that L-arg improves insulin sensitivity and modulates glucose metabolism in obesity and diabetes models [35,36]. It enhances glucose uptake in muscle and adipose tissue via NO-mediated pathways and downregulates inflammatory cytokines that impair insulin signaling. Notably, inhibition of NOS, such as by ADMA, reduces the availability and impairs insulin release, underscoring the importance of the pathway in maintaining glucose homeostasis [37]. In addition, L-arg functions as a potent insulin secretagogue, linking L-arg metabolism to glucose homeostasis through direct modulation of β-cell excitability and secretory function. Upon entering pancreatic β-cells via mCAT2A transporters, L-arg induces electrogenic depolarization of the plasma membrane. This depolarization opens voltage-gated calcium channels, allowing a Ca^2+^ influx into the cytosol. The elevated Ca^2+^ concentration triggers the exocytosis of insulin-containing secretory granules, thereby increasing insulin secretion into the bloodstream [38].

Besides its role in glucose metabolism, L-arg also influences lipid metabolism. One key mechanism involves the activation of AMP-activated protein kinase (AMPK), which inhibits acetyl-CoA carboxylase (ACC), leading to a reduction in malonyl-CoA levels and a subsequent decrease in fatty acid synthesis. Additionally, L-arg supplementation modulates the expression of sterol regulatory element-binding protein-1c (SREBP-1c), stearoyl Co-A desaturase-1 (SCD1), fatty acid synthase (FAS), and acetyl Co-A carboxylase (ACC), transcription factors that regulate genes involved in lipogenesis [39]. Through these mechanisms, L-arg contributes to reduced lipid accumulation, enhanced fatty acid oxidation, and improved lipid homeostasis, making it a potential therapeutic agent in conditions like obesity, dyslipidemia, and metabolic syndrome. Consistently, L-arg supplementation has been shown to reduce white adipose tissue (WAT) mass and modulate the ratio between brown adipose tissue (BAT) and WAT, suggesting a potential role for L-arg in promoting metabolic remodeling and enhancing energy expenditure through BAT activation [40,41,42].

Arginine metabolism also affects mitochondrial function and redox homeostasis. In hepatocytes, L-arg is converted by arginase to ornithine and urea; ornithine can enter mitochondria and feed into the TCA cycle via glutamate and α-ketoglutarate, supporting oxidative phosphorylation and cellular redox balance [22]. L-arg also participates in creatine synthesis via AGAT and GAMT, producing phosphocreatine, which buffers ATP supply and helps meet mitochondrial energy demands. Through NOS, L-arg produces NO, which can reversibly inhibit cytochrome c oxidase (complex IV) in the electron transport chain, adjusting ATP production, especially under hypoxic or inflammatory conditions. In redox regulation, NO interacts with reactive oxygen species to influence oxidative stress, while L-arg-derived ornithine serves as a precursor for polyamines that stabilize mitochondrial membranes and modulate oxidative stress–sensitive signaling. Additionally, ornithine can be transaminated to glutamate and proline. Glutamate is a key precursor for glutathione, one of the cell’s major antioxidants, further linking L-arg metabolism to the maintenance of redox balance and mitochondrial integrity [22,43].

However, some studies involving diseased subjects and prospective studies with healthy individuals have found that higher dietary L-arg intake is associated with worsening of an existing disease or may be a risk factor for the development of some diseases such as coronary heart disease [44] and type 2 diabetes mellitus [45]. The inconsistent outcome may result, among other reasons, from potential confounding variables present in a particular experimental setting, differences in the experimental models used, doses, and duration of treatment. The mechanisms underlying L-arg’s regulatory effects on carbohydrate and lipid metabolism are not yet fully understood and are currently under investigation.

## 3. Metabolic Functions of Arginine in Physiological Angiogenesis

**Figure 2:**
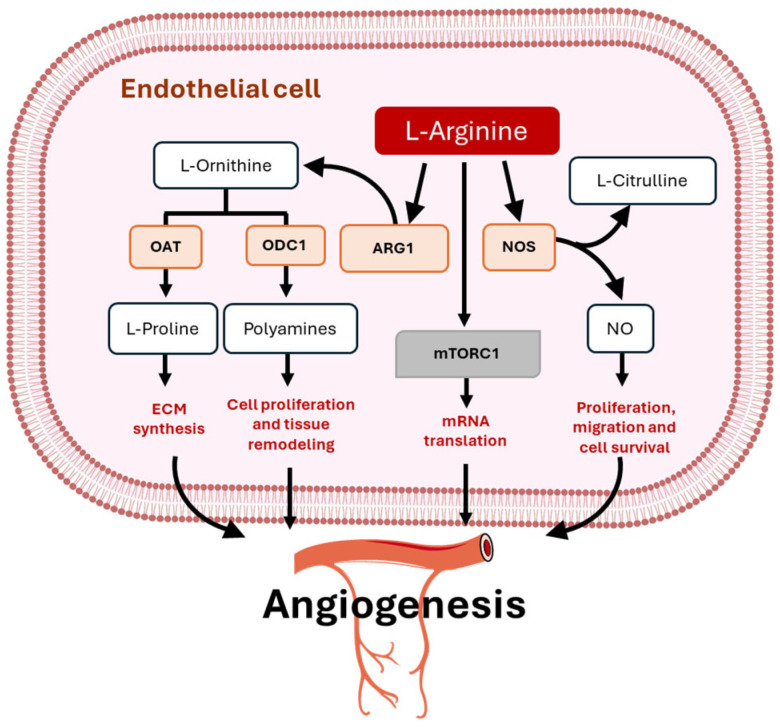
Role of L-arginine metabolism in angiogenesis. L-arginine (L-arg) serves as a substrate for NOS, which specifically catalyzes the conversion of L-arg to L-citrulline and NO, thereby promoting EC proliferation, migration, and survival. Meanwhile, arginase converts L-arg to urea and L-ornithine that is further metabolized by ODC1 to polyamines, which play a significant role in cell proliferation and tissue remodeling. L-ornithine can also be converted by OAT to L-proline, essential for collagen formation. L-arg is able to activate mTORC1, thus controlling mRNA translation. Abbreviations: ODC1—ornithine decarboxylase 1, OAT—ornithine aminotransferase, NOS—nitric oxide synthase, NO—nitric oxide, ARG1—arginase 1, and mTORC1—mammalian target of rapamycin complex 1. BioRender was used to generate the illustration.

Arginine plays a pivotal role in physiological angiogenesis, primarily as a precursor of NO, a potent vasodilator and key regulator of EC function and the vascular homeostasis. There are three isoforms of NOS: neuronal (nNOS), inducible (iNOS), and endothelial (eNOS). All NOS isoforms use L-arg as a substrate, along with oxygen, NADPH, and BH4, to generate NO and citrulline. NO promotes the proliferation, migration, and survival of ECs, which are essential processes for the formation of new blood vessels. This mechanism is particularly important for maintaining a healthy cardiovascular system and during wound healing, tissue regeneration, and angiogenesis, where sufficient blood supply is critical [46,47] (Figure 2). However, the dysfunction of the eNOS enzyme is intricately correlated with the pathogenesis of several cardiovascular diseases such as hypertension, arteriosclerosis, myocardial infarction, and stroke [48,49].

Arginine is a central substrate for eNOS, enabling NO production that governs vasodilation, angiogenesis, and vascular homeostasis. Experimental evidence from animal models demonstrates that L-arg supplementation enhances angiogenic capacity through the increased expression of vascular endothelial growth factor (VEGF) and Angiopoietin-1 (Ang-1) [50,51]. In addition, in retinal ECs, supplementation with citrulline plus L-arg has been shown to boost NO levels, which in turn enhances angiogenic sprouting and increases permeability [52]. The angiogenic role of L-arg is tightly regulated by its intracellular transporters, such as CAT-1 (SLC7A1), directly coupling extracellular L-arg availability to NO synthesis and angiogenic capacity [18]. In ECs, CD98hc (SLC3A2) forms complexes with y^+^LAT1/2 (SLC7A6/7), facilitating the L-arg import essential for NO production and vascular function. The disruption of CD98hc trafficking, as observed in ataxia telangiectasia models, impairs amino acid transport and exacerbates endothelial dysfunction, underlining its significance for vascular health [53].

In contrast, reduction in L-arg levels diminishes NO production, a critical mediator of EC migration, survival, and vascular network formation. Indeed, the inhibition of NO synthesis disrupts downstream signaling pathways, including cyclic guanosine monophosphate (cGMP) generation and VEGF-mediated responses, ultimately leading to reduced capillary density and impaired tissue perfusion [50]. The dimethylarginine dimethylaminohydrolase (DDAH)/asymmetric dimethylarginine (ADMA) pathway plays a pivotal role in regulating NO bioavailability and angiogenesis. ADMA is a product of post-translational methylation of L-arg residues and subsequent protein turnover, and it is an endogenous competitive inhibitor of NOS. By limiting the conversion of L-arg into NO and citrulline, elevated ADMA levels contribute to endothelial dysfunction, impaired angiogenesis, and the pathogenesis of cardiovascular and metabolic diseases [54,55]. Finally, the restoration of NO levels, through increased L-arg availability or by inhibiting arginase to reduce substrate competition, has been shown to improve endothelial function and enhance angiogenic capacity [48,49].

Moreover, L-arg metabolism via arginase leads to the production of ornithine (Figure 2), which is further converted into polyamines such as putrescine, spermidine, and spermine and proline. Polyamines are essential for cell proliferation and tissue remodeling, processes that are integral to angiogenesis [56]. On the other hand, proline contributes to angiogenesis primarily through its structural role in extracellular matrix (ECM) synthesis. Proline is a vital amino acid in angiogenesis due to its incorporation into collagen, a key structural component of the ECM that forms the scaffold for new blood vessels [57,58]. In addition, post-translational arginylation of β-actin plays a critical role in regulating EC behavior during angiogenesis. By enzymatically adding L-arg residues to β-actin, this modification modulates cytoskeletal dynamics, enhancing filament remodeling and cellular flexibility. These changes are essential for directed EC migration, a key step in new blood vessel formation [59]. Excessive arginase activity can compete with eNOS for L-arg, reducing NO production and impairing angiogenic signaling. L-arg depletion, whether through enzymatic degradation by L-arg deiminase or excessive arginase activity, has been shown to impair angiogenesis by limiting substrate availability for eNOS. In addition, arginase activity serves as a crucial modulator of NO bioavailability under hypoxic conditions. In vitro studies using bovine aortic ECs revealed that arginase inhibition restored NO levels, increased VEGF production, reduced reactive oxygen species, and rescued angiogenic network formation during hypoxia [60,61]. Increased arginase activity during oxygen deprivation limits L-arg availability for eNOS, impairing NO-mediated angiogenesis. This metabolic competition between arginase and NOS for L-arg underscores the importance of controlling arginase activity to preserve vascular growth capacity in ischemic tissues.

Recent advances in biomaterials have further demonstrated the therapeutic potential of L-arg. Kazemi et al. developed core–shell nanofibers that released L-arg in a controlled manner, significantly enhancing angiogenesis and granulation tissue formation in full-thickness dermal wounds [62]. The elevated local NO levels increased VEGF expression, one of the most important drivers of neovascularization. Similarly, Hussein et al. demonstrated that L-arg-loaded electrospun nanofibers improved endothelial activity, enhanced cell proliferation, and promoted wound gap closure within 48 h in vitro [63].

## 4. Arginine and Tissue Repair: Unlocking New Therapeutic Horizons

Arginine is a crucial mediator in tissue repair driving mechanisms like angiogenesis, collagen deposition, and immune modulation.

A meta-analysis published in 2021 has demonstrated that L-arg supplementation improves wound healing markers in humans. Notably, it was associated with increased hydroxyproline content, a biochemical marker of collagen deposition, highlighting its role in ECM remodeling [64]. In addition to structural protein synthesis, L-arg has been shown to interact with growth hormone and insulin-like growth factor 1 (IGF-1) pathways. These anabolic hormones play a critical role in stimulating fibroblast activity, enhancing protein deposition, and promoting cellular proliferation in damaged tissues. L-arg supplementation can increase IGF-1 levels, creating a systemic and local environment favorable to tissue regeneration [65]. In addition, the therapeutic potential of L-arg in wound healing has been closely examined with respect to optimal dosing strategies. Clinical evidence indicates that daily supplementation with 4.5–9 g of L-arg effectively supports wound closure in both malnourished and adequately nourished patients [66]. Even though a recent meta-analysis proposed that higher doses (>15 g/day) may offer additional benefits, these findings were based on only two small trials with short follow-up periods (2–3 weeks) and limited sample sizes, underscoring the need for more robust studies to clarify dose–response effects [67].

After tissue injury, cells regulate global and selective mRNA translation [68]. The mRNA translation is swiftly enhanced to produce the proteins that are essential for regeneration. This boost in protein synthesis is closely linked to mTORC1 activation, associated with ribosome biogenesis. Recent studies reveal that various signaling pathways, RNA-binding proteins, and RNA modifications play critical roles in orchestrating this process during tissue repair. Also, L-arg methylation, a post-translational modification, has been implicated in regulating mRNA translation [69,70]. It has been reported that L-arg directly regulates mTORC1 activity via suppression of TSC2’s lysosomal localization, promoting Rheb-mediated activation of mTORC1 [71]. By engaging L-arg sensors such as CASTOR1 and SLC38A9, L-arg availability activates mTORC1, which in turn promotes the translation of mRNAs encoding ribosomal proteins, growth factors, and matrix-related proteins essential for wound closure. In ECs, this mechanism ensures that protein synthesis is coupled to nutrient supply and vascular demands, linking metabolism with angiogenic capacity. In addition to mTOR-driven mechanisms, post-translational modifications such as L-arg methylation influence RNA-binding proteins that selectively modulate mRNA fate. These modifications affect the stability, localization, and translational efficiency of transcripts involved in stress responses, cytoskeletal remodeling, and ECM deposition [72]. Clinical and experimental evidence further supports these mechanisms. For example, L-arg supplementation has been shown to accelerate wound healing in surgical patients with chronic ulcers by improving nitrogen balance and enhancing immune responses [73,74]. However, while clinical and experimental evidence supports L-arg’s beneficial effects on angiogenesis, collagen deposition, immune response, and wound healing, the potential connection between L-arg and mRNA regulation in the context of tissue repair remains unclear and warrants further investigation.

Collectively, the evidence demonstrates that L-arg improves multiple aspects of wound healing by promoting collagen deposition, enhancing angiogenesis, and strengthening the mechanical properties of repaired tissue.

## 5. Arginine as Tumor Angiogenic Driver

**Figure 3:**
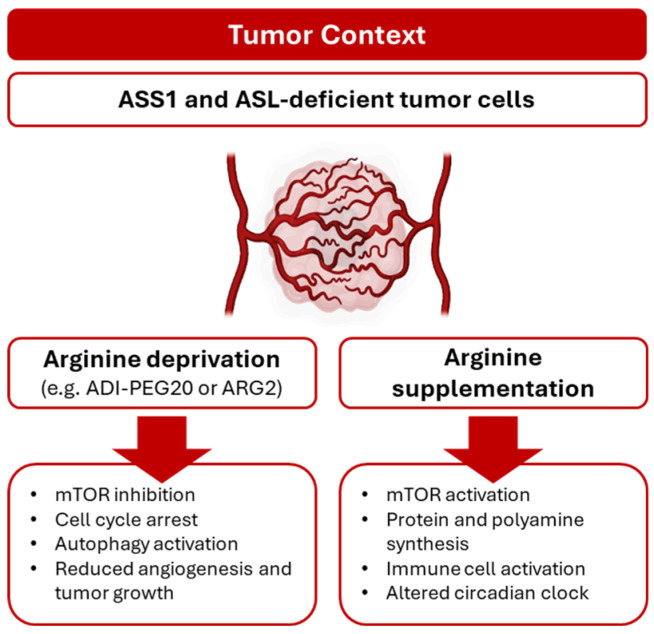
Role of L-arg metabolism in tumors and its crosstalk with angiogenesis. Arginine deprivation, particularly via ADI-PEG20, represents a promising therapeutic strategy by targeting tumors with ASS1 or ASL silencing and impairing both tumor growth and angiogenesis. While L-arg supplementation exerts a protective effect on vascular function and immune response.

Arginine deprivation has emerged as a promising metabolic therapeutic strategy in cancer treatment due to the auxotrophy of certain tumors [22]. This dependency on L-arg is typically caused by the silencing of ASS1 or ASL genes, rendering these tumors vulnerable to L-arg limitation (Figure 3). A range of malignancies, including melanoma, glioblastoma, and pancreatic, colorectal, and hepatocellular carcinomas, are characterized by a reduced expression of ASS1, resulting in a dependence on extracellular L-arg [75]. This metabolic dependency can be therapeutically exploited using arginine-degrading enzymes, which selectively inhibit tumor growth while sparing normal cells that retain endogenous L-arg synthesis. Among these agents, bacterial-derived arginine deiminase (ADI), particularly its stable and less immunoreactive PEGylated form, ADI-PEG20, has gained prominence and is currently under clinical investigation in different tumors [76,77,78]. Furthermore, ADI-PEG20 has been shown to suppress angiogenic processes in vitro, including capillary-like tube formation, and in vivo, as demonstrated in the chick chorioallantoic membrane model and Matrigel plug assays [79]. These findings suggest a significant contribution of extracellular L-arg to EC fitness. However, ADI-PEG20’s role in modulating tumor angiogenesis remains insufficiently explored and warrants further investigation.

The ASS1-deficient cancer cells, with their high demand for L-arg and inability to synthesize it, enter a starvation state and become cytostatic. Therefore, they must adapt metabolically to survive or die. In this context, various molecular mechanisms of resistance have been described. In the short term, intracellular L-arg depletion inhibits mTORC1 signaling and activates autophagy [80], which recycles intracellular L-arg to sustain cellular viability. In fact, the co-treatment of ASS1-deficient tumors with chloroquine, an inhibitor of autophagy, enhanced its effect [81]. Studies have shown that, in order to achieve long-term resistance to L-arg deprivation therapy, cancer cells may upregulate ASS1 expression in a MYC-dependent manner [82]. Alternatively, they may activate compensatory mechanisms to restore L-arg levels, such as macropinocytosis, endocytosis, phagocytosis, entosis, extracellular vesicle uptake, nutrient exchange via gap junctions, and L-arg secretion by neighboring cells [83]. These diverse strategies offer cancer cells multiple avenues to circumvent L-arg starvation and maintain growth. Emerging evidence also suggests that ECs within the tumor microenvironment may contribute to L-arg availability by releasing L-arg-rich metabolites or facilitating nutrient exchange through specialized junctions [75,84]. However, it remains unclear whether ECs can adapt to L-arg limitation in either acute or chronic settings, as well as the mechanisms underlying such adaptation in tumor contexts.

Cellular L-arg availability is also determined, in part, by the efficiency and capacity of L-arg transporters expressed in the plasma membrane [85]. Human ECs use two specialized systems to transport L-arg across their cell membranes. The first system is called system y^+^L, which can be blocked by leucine. This system is formed by two subunits: a heavy subunit called 4F2hc/CD98, and a light subunit that can be either y^+^LAT1 or y^+^LAT2 (SLC7A7 or SLC7A6, respectively). The second system, system y^+^, is sensitive to N-ethylmaleimide (NEM), but it works even when leucine is present. This system is formed by a single subunit from the CAT family. The most common one is the cationic amino acid transporter-1 (CAT1) protein, found throughout the body and encoded by the *SLC7A1* gene. The other two, CAT2A and CAT2B, come from the *SLC7A2* gene and are produced by alternative splicing. CAT2A has a lower ability to bind L-arg, while CAT2B binds it more strongly [86]. In cultured ECs, 70–95% of extracellular L-arg uptake has been attributed to CAT1 [87].

Dysregulation of L-arg transport in ECs has been linked to endothelial dysfunction, a hallmark of several pathologies including cancer, where altered NO signaling can influence tumor progression and vascular remodeling [88]. Interestingly, extracellular vesicles (EVs) derived from human colorectal cancer cells present high levels of CAT1 that can be transferred to vascular ECs and promote angiogenesis by altering NO metabolism. Indeed, ECs cultured with CAT1-EVs showed an increase in proliferation and tube formation [89]. A comparative study has shown that tumor necrosis factor-α (TNFα), a known cytokine involved in all stages of the tumor malignant process [90], stimulates system y+-mediated L-arg uptake in saphenous and umbilical vein human ECs. These changes are associated with an increase in the intracellular L-arg concentration but with a decrease in NO production [91]. Although these transporters are dynamically regulated in ECs, whether their modulation could be exploited to block or normalize tumor vasculature remains an open and unexplored therapeutic strategy. Furthermore, Ong et al. have described that retinal ECs activate the YAP/TAZ–TEAD pathway to support retinal angiogenesis by controlling amino acid transporters, including CAT1, which are necessary to activate mTORC1 to support mRNA translation [92,93].

The angiogenic process is controlled at mRNA translational levels [94]. Among amino acids, L-arg plays a particularly important role in modulating translation. Uniquely, L-arg binds to CASTOR1, disrupting its interaction with GATOR2, a negative regulator of the mTORC1 pathway. As a result, mTORC1 activation by L-arg depends on CASTOR1’s L-arg-binding capacity, positioning CASTOR1 as a key L-arg sensor within the mTORC1 signaling axis [95]. Once activated, mTORC1 orchestrates several aspects of mRNA translation, including cap-dependent translation via phosphorylation of 4EBPs and S6K1, translation of 5′-TOP mRNAs, transcription of ribosome biogenesis (RiBi) genes through S6K1, and ribosome assembly via phosphorylation of UBF and TIF1A in the nucleolus and Maf1 in the nucleus [96].

Under conditions of L-arg scarcity, selective loss of L-arg tRNA charging leads to ribosome pausing at specific L-arg codons, thereby impairing translation. In contrast, leucine limitation, despite leucine being an abundant and essential amino acid, induces little to no ribosome pausing. This differential response is attributed to the robust activation of mTORC1 and GCN2 signaling under leucine deprivation, which prevents pausing, whereas L-arg limitation elicits a weaker response [97].

During nutrient limitation, amino acid misincorporation can occur through codon-anticodon mispairing, effectively promoting codon reassignment. In colorectal cancer, L-arg deprivation disrupts the translation of L-arg codons, induces a shift toward an L-arg-low proteome, and drives codon-specific cancer evolution with preferential mutation of L-arg codons to other amino acids such as cysteine or histidine [98]. Similarly, Yang et al. reported an enrichment of L-arg-to-cysteine substitutions in a subset of lung cancer proteomes, which is potentiated by L-arg deprivation and promotes resistance to chemotherapy [99,100]. Although the role of certain amino acids in regulating translation during angiogenesis has been reported [101], the specific impact of L-arg on the translational machinery within the context of angiogenesis remains largely unexplored.

It is noteworthy that glucose starvation and hypoxia, hallmark features of tumor microenvironment, reprogram L-arg metabolism in ECs. Indeed, HUVECs exposed to glucose limitation and hypoxia downregulate ODC1 and ASS1 while upregulating ARG2 and spermidine-spermine acetyltransferase 1 (SAT1) [102]. These results reflect a metabolic shift from polyamine synthesis toward catabolism [103], along with a decline in de novo L-arg biosynthesis, suggesting a transition toward arginine auxotrophy in the tumor context. Additional experiments conducted in microvascular ECs revealed a rewiring of amino acid metabolism, including L-arg, under both acute and chronic hypoxia [104].

The circadian rhythm is an endogenous clock system that coordinates and optimizes various physiological and pathophysiological processes, including angiogenesis [105] and cancer [106]. Recently, it has been described that circadian locomotor output cycles kaput (CLOCK) directly acetylates the K165 and K176 residues of ASS1, leading to the rhythmic inactivation of ASS1. CLOCK-induced inactivation of ASS1 displays circadian oscillation in human cells and mouse liver, possibly resulting from rhythmic interaction between CLOCK and ASS1 and thereby regulating the circadian rhythm of ASS1 and ureagenesis [107]. Hu et al. showed that L-arg (with methionine) supplementation alters CLOCK and PER1 protein abundance, influencing mTORC1 signaling and α-s1-casein synthesis [108] (Figure 3). Furthermore, Long et al. demonstrate that, in human cancer cell lines, cisplatin suppresses ASS1 expression via a network involving DEC1, HIF-1α, and c-Myc [109]. Conversely, high levels of ASS1 have been shown to play a protective role in vascular function [110]. Additionally, an ASS1 increase in ECs has been observed in tumor contexts, including breast cancer [111]. Overall, the intricate regulation of ASS1 by the circadian clock underscores its pivotal role in synchronizing metabolic and physiological processes such as ureagenesis, vascular function, and tumor biology. The dynamic interplay between CLOCK and ASS1 not only highlights the importance of temporal control in cellular metabolism but also reveals potential therapeutic targets for cancer and vascular disorders. Understanding how circadian rhythms influence ASS1 activity could pave the way for novel interventions that harness the body’s internal clock to optimize treatment outcomes and maintain vascular health.

## 6. Clinical Implications of Arginine in Tumor Angiogenesis

Arginine metabolism represents a promising target in cancer therapy due to its critical role in tumor growth, immune modulation, and angiogenesis. Preclinical studies have demonstrated that arginine deprivation can inhibit tumor proliferation, induce apoptosis, and reduce angiogenic signaling. Strategies such as arginine deiminase and human arginase (hArg) exploit arginine auxotrophy in tumors, particularly those with deficiencies in arginosuccinate synthesis 1 (ASS1) or ornithine transcarbamylase (OCT), including hepatocellular carcinoma and melanoma. However, translating these promising mechanisms into clinical success remains challenging.

Pharmacokinetic limitations and immunogenicity are significant barriers for arginine-degrading enzymes. ADI modified with polyethylene glycol and hArg have demonstrated potential in early clinical trials, yet their short half-lives, immunogenic responses, and batch-to-batch variability constrain therapeutic efficacy [112,113]. Efforts in protein engineering, including PEGylation and fusion with human serum albumin, aim to enhance stability, prolong circulation, and reduce immunogenicity, but clinical validation is ongoing [114,115].

Safety and specificity are additional concerns. Inhibitors of dimethylarginine dimethylaminohydrolase 1 (DDAH1), a key regulator of nitric oxide synthase, remain in the preclinical stage, with unresolved risks of off-target effects and cardiovascular toxicity [116]. The development of robust assays for DDAH2 and the verification of inhibitor specificity are necessary before broader clinical application can be considered.

Patient selection presents another translational challenge due to the heterogeneity of arginine dependency across tumor types. While biomarker-driven approaches based on ASS1 or OTC deficiencies provide a rationale for targeted therapy, standardized selection protocols are lacking. Furthermore, tumors can develop resistance through upregulation of ASS1 or activation of compensatory metabolic pathways, limiting the long-term efficacy of arginine-targeted interventions.

Combination strategies offer a potential avenue to overcome resistance and enhance efficacy. Early studies combining ADI or hArg with chemotherapy or immunotherapy suggest synergistic effects, but optimal dosing, timing, and sequencing remain to be defined [113]. Overall, although preclinical and early clinical evidence supports the promise of arginine-targeting approaches, the translation into clinical practice is constrained by pharmacologic limitations, safety concerns, tumor heterogeneity, and lack of high-quality, large-scale clinical trials with standardized biomarkers.

Collectively, these findings underscore that, while arginine-targeting strategies hold significant potential in both the metabolic and the angiogenic regulation of tumors, successful clinical translation will require integrated solutions, combining precise patient stratification, advanced protein engineering, and rational combination therapies.

## 7. Remarkable Conclusions

Arginine is essential for supporting various functional aspects of angiogenesis, either directly or through its downstream metabolites. Additionally, it participates in other metabolic pathways, including glutaminolysis, proline and spermidine synthesis, ureagenesis, and NO production. This makes L-arg an intriguing target for modulating tumor angiogenesis as well as for use in tissue regeneration processes. Importantly, the role of arginine in tumor progression and vascular normalization warrants deeper investigation, as it may reveal critical mechanisms by which tumor vasculature adapts or can be therapeutically normalized to improve treatment outcomes. Despite these insights, many questions remain unanswered, particularly regarding how L-arg flux is regulated during tumor versus physiological angiogenesis and whether this regulation is preserved in a tumor- or organ-specific manner. To explore these mechanisms with greater precision, techniques such as MALDI mass spectrometry imaging have been developed; however, enhancements in sensitivity at the single-cell mass spectrometry level are still required. Moreover, reprogramming of the translational machinery in response to amino acid deprivation has been documented in tumor environments, resulting in a proteome overhaul. Yet, whether ECs exhibit a similar adaptive response remains unexplored. Overall, advancing our understanding of L-arg metabolism in angiogenesis will continue to be a vital focus within vascular research, fueling new insights and breakthroughs in endothelial cell biology.

## Data Availability

No new data were created or analyzed in this study.

## References

[1] Eelen G., Treps L., Li X., Carmeliet P. (2020). Basic and Therapeutic Aspects of Angiogenesis Updated. Circ. Res..

[2] Augustin H.G., Koh G.Y. (2024). A systems view of the vascular endothelium in health and disease. Cell.

[3] Luo Z., Yao J., Wang Z., Xu J. (2023). Mitochondria in endothelial cells angiogenesis and function: Current understanding and future perspectives. J. Transl. Med..

[4] Siekmann A.F. (2023). Biology of vascular mural cells. Dev. Camb. Engl..

[5] Han C., Barakat M., DiPietro L.A. (2022). Angiogenesis in Wound Repair: Too Much of a Good Thing?. Cold Spring Harb. Perspect. Biol..

[6] Chen H., Peng C., Fang F., Li Y., Liu X., Hu Y., Wang G., Liu X., Shen Y. (2025). Angiogenesis within atherosclerotic plaques: Mechanical regulation, molecular mechanism and clinical diagnosis. Mechanobiol. Med..

[7] Fadini G.P., Albiero M., Bonora B.M., Avogaro A. (2019). Angiogenic Abnormalities in Diabetes Mellitus: Mechanistic and Clinical Aspects. J. Clin. Endocrinol. Metab..

[8] Corvera S., Solivan-Rivera J., Yang Loureiro Z. (2022). Angiogenesis in adipose tissue and obesity. Angiogenesis.

[9] Carmeliet P., Jain R.K. (2000). Angiogenesis in cancer and other diseases. Nature.

[10] Cantelmo A.R., Conradi L.-C., Brajic A., Goveia J., Kalucka J., Pircher A., Chaturvedi P., Hol J., Thienpont B., Teuwen L.-A. (2016). Inhibition of the Glycolytic Activator PFKFB3 in Endothelium Induces Tumor Vessel Normalization, Impairs Metastasis, and Improves Chemotherapy. Cancer Cell.

[11] De Bock K., Georgiadou M., Schoors S., Kuchnio A., Wong B.W., Cantelmo A.R., Quaegebeur A., Ghesquière B., Cauwenberghs S., Eelen G. (2013). Role of PFKFB3-driven glycolysis in vessel sprouting. Cell.

[12] Kalucka J., Bierhansl L., Conchinha N.V., Missiaen R., Elia I., Brüning U., Scheinok S., Treps L., Cantelmo A.R., Dubois C. (2018). Quiescent Endothelial Cells Upregulate Fatty Acid β-Oxidation for Vasculoprotection via Redox Homeostasis. Cell Metab..

[13] Bruning U., Morales-Rodriguez F., Kalucka J., Goveia J., Taverna F., Queiroz K.C.S., Dubois C., Cantelmo A.R., Chen R., Loroch S. (2018). Impairment of Angiogenesis by Fatty Acid Synthase Inhibition Involves mTOR Malonylation. Cell Metab..

[14] Kim B., Li J., Jang C., Arany Z. (2017). Glutamine fuels proliferation but not migration of endothelial cells. EMBO J..

[15] Huang H., Vandekeere S., Kalucka J., Bierhansl L., Zecchin A., Brüning U., Visnagri A., Yuldasheva N., Goveia J., Cruys B. (2017). Role of glutamine and interlinked asparagine metabolism in vessel formation. EMBO J..

[16] Upadhyayula P.S., Higgins D.M., Mela A., Banu M., Dovas A., Zandkarimi F., Patel P., Mahajan A., Humala N., Nguyen T.T.T. (2023). Dietary restriction of cysteine and methionine sensitizes gliomas to ferroptosis and induces alterations in energetic metabolism. Nat. Commun..

[17] Lermant A., Murdoch C.E. (2019). Cysteine Glutathionylation Acts as a Redox Switch in Endothelial Cells. Antioxidants.

[18] Kurhaluk N., Tkaczenko H. (2025). L-Arginine and Nitric Oxide in Vascular Regulation—Experimental Findings in the Context of Blood Donation. Nutrients.

[19] Carlström M. (2021). Nitric oxide signalling in kidney regulation and cardiometabolic health. Nat. Rev. Nephrol..

[20] Molek P., Zmudzki P., Wlodarczyk A., Nessler J., Zalewski J. (2021). The shifted balance of arginine metabolites in acute myocardial infarction patients and its clinical relevance. Sci. Rep..

[21] Wu G., Meininger C.J. (2000). Arginine Nutrition and Cardiovascular Function. J. Nutr..

[22] Fung T.S., Ryu K.W., Thompson C.B. (2025). Arginine: At the crossroads of nitrogen metabolism. EMBO J..

[23] Janaszak-Jasiecka A., Płoska A., Wierońska J.M., Dobrucki L.W., Kalinowski L. (2023). Endothelial dysfunction due to eNOS uncoupling: Molecular mechanisms as potential therapeutic targets. Cell. Mol. Biol. Lett..

[24] Bode-Böger S.M., Scalera F., Ignarro L.J. (2007). The L-arginine paradox: Importance of the L-arginine/asymmetrical dimethylarginine ratio. Pharmacol. Ther..

[25] Starikova E.A., Rubinstein A.A., Mammedova J.T., Isakov D.V., Kudryavtsev I.V. (2023). Regulated Arginine Metabolism in Immunopathogenesis of a Wide Range of Diseases: Is There a Way to Pass between Scylla and Charybdis?. Curr. Issues Mol. Biol..

[26] Kieler M., Hofmann M., Schabbauer G. (2021). More than just protein building blocks: How amino acids and related metabolic pathways fuel macrophage polarization. FEBS J..

[27] Locke M., Ghazaly E., Freitas M.O., Mitsinga M., Lattanzio L., Lo Nigro C., Nagano A., Wang J., Chelala C., Szlosarek P. (2016). Inhibition of the Polyamine Synthesis Pathway Is Synthetically Lethal with Loss of Argininosuccinate Synthase 1. Cell Rep..

[28] Nakanishi S., Cleveland J.L. (2021). Polyamine Homeostasis in Development and Disease. Med. Sci. Basel Switz..

[29] Tauer K., Theile C., Owens J.W., Cecil K.M., Shillington A. (2024). Arginine, glycine, and creatine supplementation improves symptoms in a female with creatine transporter deficiency. Psychiatr. Genet..

[30] Diaz G.A., Bechter M., Cederbaum S.D. (2023). The role and control of arginine levels in arginase 1 deficiency. J. Inherit. Metab. Dis..

[31] Rabinovich S., Adler L., Yizhak K., Sarver A., Silberman A., Agron S., Stettner N., Sun Q., Brandis A., Helbling D. (2015). Diversion of aspartate in ASS1-deficient tumours fosters de novo pyrimidine synthesis. Nature.

[32] Saxton R.A., Sabatini D.M. (2017). mTOR Signaling in Growth, Metabolism, and Disease. Cell.

[33] Takahara T., Amemiya Y., Sugiyama R., Maki M., Shibata H. (2020). Amino acid-dependent control of mTORC1 signaling: A variety of regulatory modes. J. Biomed. Sci..

[34] Panwar V., Singh A., Bhatt M., Tonk R.K., Azizov S., Raza A.S., Sengupta S., Kumar D., Garg M. (2023). Multifaceted role of mTOR (mammalian target of rapamycin) signaling pathway in human health and disease. Signal Transduct. Target. Ther..

[35] Forzano I., Avvisato R., Varzideh F., Jankauskas S.S., Cioppa A., Mone P., Salemme L., Kansakar U., Tesorio T., Trimarco V. (2023). L-Arginine in diabetes: Clinical and preclinical evidence. Cardiovasc. Diabetol..

[36] Hu S., Han M., Rezaei A., Li D., Wu G., Ma X. (2017). L-Arginine Modulates Glucose and Lipid Metabolism in Obesity and Diabetes. Curr. Protein Pept. Sci..

[37] Guo X., Xing Y., Jin W. (2023). Role of ADMA in the pathogenesis of microvascular complications in type 2 diabetes mellitus. Front. Endocrinol..

[38] White P.J., Wewer Albrechtsen N.J., Campbell J.E. (2025). Islet hormones at the intersection of glucose and amino acid metabolism. Nat. Rev. Endocrinol..

[39] Ali A., Liu X., Melaku M., Lqbal W., Yi B., Zhong R., Chen L., Ma T., Zhang H. (2025). Effect of arginine supplementation on liver and pectoral muscle: Tissue-specific lipid metabolism in broilers. Poult. Sci..

[40] McKnight J.R., Satterfield M.C., Jobgen W.S., Smith S.B., Spencer T.E., Meininger C.J., McNeal C.J., Wu G. (2010). Beneficial effects of L-arginine on reducing obesity: Potential mechanisms and important implications for human health. Amino Acids.

[41] Kalezic A., Korac A., Korac B., Jankovic A. (2022). l-Arginine Induces White Adipose Tissue Browning-A New Pharmaceutical Alternative to Cold. Pharmaceutics.

[42] Jobgen W.S., Lee M.-J., Fried S.K., Wu G. (2023). l-Arginine supplementation regulates energy-substrate metabolism in skeletal muscle and adipose tissue of diet-induced obese rats. Exp. Biol. Med..

[43] Wu G., Morris S.M. (1998). Arginine metabolism: Nitric oxide and beyond. Biochem. J..

[44] Bahadoran Z., Mirmiran P., Tahmasebinejad Z., Azizi F. (2016). Dietary L-arginine intake and the incidence of coronary heart disease: Tehran lipid and glucose study. Nutr. Metab..

[45] Mirmiran P., Bahadoran Z., Gaeini Z., Azizi F. (2021). Habitual intake of dietary L-arginine in relation to risk of type 2 diabetes: A prospective study. BMC Endocr. Disord..

[46] Smith T.L., Oubaha M., Cagnone G., Boscher C., Kim J.S., El Bakkouri Y., Zhang Y., Chidiac R., Corriveau J., Delisle C. (2021). eNOS controls angiogenic sprouting and retinal neovascularization through the regulation of endothelial cell polarity. Cell. Mol. Life Sci..

[47] Tran N., Garcia T., Aniqa M., Ali S., Ally A., Nauli S.M. (2022). Endothelial Nitric Oxide Synthase (eNOS) and the Cardiovascular System: In Physiology and in Disease States. Am. J. Biomed. Sci. Res..

[48] Roy R., Wilcox J., Webb A.J., O’Gallagher K. (2023). Dysfunctional and Dysregulated Nitric Oxide Synthases in Cardiovascular Disease: Mechanisms and Therapeutic Potential. Int. J. Mol. Sci..

[49] Leiper J., Nandi M. (2011). The therapeutic potential of targeting endogenous inhibitors of nitric oxide synthesis. Nat. Rev. Drug Discov..

[50] Ranjbar K. (2022). Improved Cardiac Function Following Ischemia Reperfusion Injury Using Exercise Preconditioning and L-Arginine Supplementation via Oxidative Stress Mitigation and Angiogenesis Amelioration. Cardiovasc. Toxicol..

[51] Elmetwally M.A., Li X., Johnson G.A., Burghardt R.C., Herring C.M., Kramer A.C., Meininger C.J., Bazer F.W., Wu G. (2022). Dietary supplementation with L-arginine between days 14 and 25 of gestation enhances NO and polyamine syntheses and the expression of angiogenic proteins in porcine placentae. Amino Acids.

[52] Warden C., Zubieta D., Brantley M.A. (2025). Citrulline Plus Arginine Induces an Angiogenic Response and Increases Permeability in Retinal Endothelial Cells via Nitric Oxide Production. Int. J. Mol. Sci..

[53] Romero J.C., Tonapi S.S., Parihar M., Loranc E., Miller H.E., Lawrence L.A., Bassani N., Robledo D.G., Cao L., Nie J. (2025). Loss of CD98HC phosphorylation by ATM impairs antiporter trafficking and drives glutamate toxicity in Ataxia telangiectasia. Nat. Commun..

[54] Fiedler L.R., Wojciak-Stothard B. (2009). The DDAH/ADMA pathway in the control of endothelial cell migration and angiogenesis. Biochem. Soc. Trans..

[55] Cefalo C.M.A., Riccio A., Fiorentino T.V., Massimino M., Mannino G.C., Succurro E., Perticone M., Sciacqua A., Andreozzi F., Perticone F. (2024). Asymmetric dimethylarginine (ADMA) is associated with reduced myocardial mechano-energetic efficiency in hypertensive subjects. Nutr. Metab. Cardiovasc. Dis..

[56] Wu G., Meininger C.J., McNeal C.J., Bazer F.W., Rhoads J.M. (2021). Role of L-Arginine in Nitric Oxide Synthesis and Health in Humans. Adv. Exp. Med. Biol..

[57] He J., Fang B., Shan S., Li Q. (2023). Mechanical stiffness promotes skin fibrosis through Piezo1-mediated arginine and proline metabolism. Cell Death Discov..

[58] Karna E., Szoka L., Huynh T.Y.L., Palka J.A. (2020). Proline-dependent regulation of collagen metabolism. Cell. Mol. Life Sci..

[59] Karakozova M., Kozak M., Wong C.C.L., Bailey A.O., Yates J.R., Mogilner A., Zebroski H., Kashina A. (2006). Arginylation of beta-actin regulates actin cytoskeleton and cell motility. Science.

[60] Kurhaluk N. (2023). The Effectiveness of L-arginine in Clinical Conditions Associated with Hypoxia. Int. J. Mol. Sci..

[61] Li Z., Wang L., Ren Y., Huang Y., Liu W., Lv Z., Qian L., Yu Y., Xiong Y. (2022). Arginase: Shedding light on the mechanisms and opportunities in cardiovascular diseases. Cell Death Discov..

[62] Kazemi N., Javad Mahalati M., Kaviani Y., Al-Musawi M.H., Varshosaz J., Soleymani Eil Bakhtiari S., Tavakoli M., Alizadeh M., Sharifianjazi F., Salehi S. (2024). Core-shell nanofibers containing L-arginine stimulates angiogenesis and full thickness dermal wound repair. Int. J. Pharm..

[63] Hussein Y., El-Fakharany E.M., Kamoun E.A., Loutfy S.A., Amin R., Taha T.H., Salim S.A., Amer M. (2020). Electrospun PVA/hyaluronic acid/L-arginine nanofibers for wound healing applications: Nanofibers optimization and in vitro bioevaluation. Int. J. Biol. Macromol..

[64] Arribas-López E., Zand N., Ojo O., Snowden M.J., Kochhar T. (2021). The Effect of Amino Acids on Wound Healing: A Systematic Review and Meta-Analysis on Arginine and Glutamine. Nutrients.

[65] Goli P., Yazdi M., Heidari-Beni M., Kelishadi R. (2022). Growth Hormone Response to L-Arginine Alone and Combined with Different Doses of Growth Hormone-Releasing Hormone: A Systematic Review and Meta-Analysis. Int. J. Endocrinol..

[66] Leigh B., Desneves K., Rafferty J., Pearce L., King S., Woodward M.C., Brown D., Martin R., Crowe T.C. (2012). The effect of different doses of an arginine-containing supplement on the healing of pressure ulcers. J. Wound Care.

[67] Cheshmeh S., Hojati N., Mohammadi A., Rahmani N., Moradi S., Pasdar Y., Elahi N. (2022). The use of oral and enteral tube-fed arginine supplementation in pressure injury care: A systematic review and meta-analysis. Nurs. Open.

[68] Amiri M., Sonenberg N., Tahmasebi S. (2025). mRNA translational control of regeneration. Curr. Opin. Genet. Dev..

[69] Blanc R.S., Richard S. (2017). Arginine Methylation: The Coming of Age. Mol. Cell.

[70] Zaccarelli-Magalhães J., Citadin C.T., Langman J., Smith D.J., Matuguma L.H., Lin H.W., Udo M.S.B. (2025). Protein arginine methyltransferases as regulators of cellular stress. Exp. Neurol..

[71] Carroll B., Maetzel D., Maddocks O.D., Otten G., Ratcliff M., Smith G.R., Dunlop E.A., Passos J.F., Davies O.R., Jaenisch R. (2016). Control of TSC2-Rheb signaling axis by arginine regulates mTORC1 activity. eLife.

[72] Wang Z., Pan Z., Adhikari S., Harada B.T., Shen L., Yuan W., Abeywardana T., Al-Hadid Q., Stark J.M., He C. (2021). m6A Deposition is Regulated by PRMT1-Mediated Arginine Methylation of METTL14 in Its Disordered C-Terminal Region. EMBO J..

[73] Sarandy M.M., Pelinsari S.M., de Souza L.M., Novaes R.D., Zanuncio V.V., Gonçalves R.V. (2023). l-arginine and l-citrulline supplementation accelerates second intention wound healing in iNOS knockout mice. J. Funct. Foods.

[74] Torsy T., Tency I., Beeckman D., Isoherranen K., Litchford M., De Vylder F. (2025). The Role of Glutamine and Arginine in Wound Healing of Pressure Ulcers: A Systematic Review. Wound Repair Regen..

[75] Ding Q., Li R., Wang Q., Yu L., Zi F. (2023). A pan-cancer analysis of the role of argininosuccinate synthase 1 in human tumors. Front. Oncol..

[76] Chan P.Y., Phillips M.M., Ellis S., Johnston A., Feng X., Arora A., Hay G., Cohen V.M.L., Sagoo M.S., Bomalaski J.S. (2022). A Phase 1 study of ADI-PEG20 (pegargiminase) combined with cisplatin and pemetrexed in ASS1-negative metastatic uveal melanoma. Pigment Cell Melanoma Res..

[77] Przystal J.M., Hajji N., Khozoie C., Renziehausen A., Zeng Q., Abaitua F., Hajitou A., Suwan K., Want E., Bomalaski J. (2018). Efficacy of arginine depletion by ADI-PEG20 in an intracranial model of GBM. Cell Death Dis..

[78] Tsai H.-J., Hsiao H.-H., Hsu Y.-T., Liu Y.-C., Kao H.-W., Liu T.-C., Cho S.-F., Feng X., Johnston A., Bomalaski J.S. (2021). Phase I study of ADI-PEG20 plus low-dose cytarabine for the treatment of acute myeloid leukemia. Cancer Med..

[79] Park I.-S., Kang S.-W., Shin Y.-J., Chae K.-Y., Park M.-O., Kim M.-Y., Wheatley D.N., Min B.-H. (2003). Arginine deiminase: A potential inhibitor of angiogenesis and tumour growth. Br. J. Cancer.

[80] Ben-Sahra I., Manning B.D. (2017). mTORC1 signaling and the metabolic control of cell growth. Curr. Opin. Cell Biol..

[81] Duysak T., Kim K., Yun M., Jeong J.-H., Choy H.E. (2024). Enhanced anti-cancer efficacy of arginine deaminase expressed by tumor-seeking Salmonella Gallinarum. Oncogene.

[82] Prudner B.C., Rathore R., Robinson A.M., Godec A., Chang S.F., Hawkins W.G., Hirbe A.C., Van Tine B.A. (2019). Arginine Starvation and Docetaxel Induce c-Myc–Driven hENT1 Surface Expression to Overcome Gemcitabine Resistance in ASS1-Negative Tumors. Clin. Cancer Res..

[83] Rogers L.C., Kremer J.C., Brashears C.B., Lin Z., Hu Z., Bastos A.C.S., Baker A., Fettig N., Zhou D., Shoghi K.I. (2023). Discovery and Targeting of a Noncanonical Mechanism of Sarcoma Resistance to ADI-PEG20 Mediated by the Microenvironment. Clin. Cancer Res..

[84] Giatromanolaki A., Harris A.L., Koukourakis M.I. (2021). The prognostic and therapeutic implications of distinct patterns of argininosuccinate synthase 1 (ASS1) and arginase-2 (ARG2) expression by cancer cells and tumor stroma in non-small-cell lung cancer. Cancer Metab..

[85] Jungnickel K.E.J., Parker J.L., Newstead S. (2018). Structural basis for amino acid transport by the CAT family of SLC7 transporters. Nat. Commun..

[86] Visigalli R., Barilli A., Parolari A., Sala R., Rotoli B.M., Bussolati O., Gazzola G.C., Dall’Asta V. (2010). Regulation of arginine transport and metabolism by protein kinase Calpha in endothelial cells: Stimulation of CAT2 transporters and arginase activity. J. Mol. Cell. Cardiol..

[87] Zani B.G., Bohlen H.G. (2005). Transport of extracellular l-arginine via cationic amino acid transporter is required during in vivo endothelial nitric oxide production. Am. J. Physiol. Heart Circ. Physiol..

[88] Banjarnahor S., Rodionov R.N., König J., Maas R. (2020). Transport of L-Arginine Related Cardiovascular Risk Markers. J. Clin. Med..

[89] Ikeda A., Nagayama S., Sumazaki M., Konishi M., Fujii R., Saichi N., Muraoka S., Saigusa D., Shimada H., Sakai Y. (2021). Colorectal Cancer–Derived CAT1-Positive Extracellular Vesicles Alter Nitric Oxide Metabolism in Endothelial Cells and Promote Angiogenesis. Mol. Cancer Res..

[90] Balkwill F. (2006). TNF-alpha in promotion and progression of cancer. Cancer Metastasis Rev..

[91] Visigalli R., Barilli A., Bussolati O., Sala R., Gazzola G.C., Parolari A., Tremoli E., Simon A., Closs E.I., Dall’Asta V. (2007). Rapamycin stimulates arginine influx through CAT2 transporters in human endothelial cells. Biochim. Biophys. Acta.

[92] Ong Y.T., Andrade J., Armbruster M., Shi C., Castro M., Costa A.S.H., Sugino T., Eelen G., Zimmermann B., Wilhelm K. (2022). A YAP/TAZ-TEAD signalling module links endothelial nutrient acquisition to angiogenic growth. Nat. Metab..

[93] Oberkersch R.E., Santoro M.M. (2022). YAP/TAZ–TEAD link angiogenesis to nutrients. Nat. Metab..

[94] Lidonnici J., Oberkersch R.E. (2024). Reciprocal Dynamics of Metabolism and mRNA Translation in Tumor Angiogenesis. Int. J. Mol. Sci..

[95] Chantranupong L., Scaria S.M., Saxton R.A., Gygi M.P., Shen K., Wyant G.A., Wang T., Harper J.W., Gygi S.P., Sabatini D.M. (2016). The CASTOR Proteins Are Arginine Sensors for the mTORC1 Pathway. Cell.

[96] Iadevaia V., Liu R., Proud C.G. (2014). mTORC1 signaling controls multiple steps in ribosome biogenesis. Semin. Cell Dev. Biol..

[97] Darnell A.M., Subramaniam A.R., O’Shea E.K. (2018). Translational Control through Differential Ribosome Pausing during Amino Acid Limitation in Mammalian Cells. Mol. Cell.

[98] Hsu D.J., Gao J., Yamaguchi N., Pinzaru A., Wu Q., Mandayam N., Liberti M., Heissel S., Alwaseem H., Tavazoie S. (2023). Arginine limitation drives a directed codon-dependent DNA sequence evolution response in colorectal cancer cells. Sci. Adv..

[99] Hsu D.J., Tavazoie S.F. (2024). Cysteine substitutants emerge in lung cancer proteomes during arginine restriction. Mol. Cell.

[100] Yang C., Pataskar A., Feng X., Montenegro Navarro J., Paniagua I., Jacobs J.J.L., Zaal E.A., Berkers C.R., Bleijerveld O.B., Agami R. (2024). Arginine deprivation enriches lung cancer proteomes with cysteine by inducing arginine-to-cysteine substitutants. Mol. Cell.

[101] Oberkersch R.E., Pontarin G., Astone M., Spizzotin M., Arslanbaeva L., Tosi G., Panieri E., Ricciardi S., Allega M.F., Brossa A. (2022). Aspartate metabolism in endothelial cells activates the mTORC1 pathway to initiate translation during angiogenesis. Dev. Cell.

[102] Wu R., Zhong J., Song L., Zhang M., Chen L., Zhang L., Qiu Z. (2023). Untargeted metabolomic analysis of ischemic injury in human umbilical vein endothelial cells reveals the involvement of arginine metabolism. Nutr. Metab..

[103] Schibalski R.S., Shulha A.S., Tsao B.P., Palygin O., Ilatovskaya D.V. (2024). The role of polyamine metabolism in cellular function and physiology. Am. J. Physiol.-Cell Physiol..

[104] Cohen E.B., Geck R.C., Toker A. (2020). Metabolic pathway alterations in microvascular endothelial cells in response to hypoxia. PLoS ONE.

[105] Astone M., Oberkersch R.E., Tosi G., Biscontin A., Santoro M.M. (2023). The circadian protein BMAL1 supports endothelial cell cycle during angiogenesis. Cardiovasc. Res..

[106] Fortin B.M., Mahieu A.L., Fellows R.C., Kang Y., Lewis A.N., Ead A.S., Lamia K.A., Cao Y., Pannunzio N.R., Masri S. (2025). The diverse roles of the circadian clock in cancer. Nat. Cancer.

[107] Lin R., Mo Y., Zha H., Qu Z., Xie P., Zhu Z.-J., Xu Y., Xiong Y., Guan K.-L. (2017). CLOCK Acetylates ASS1 to Drive Circadian Rhythm of Ureagenesis. Mol. Cell.

[108] Hu L., Chen Y., Cortes I.M., Coleman D.N., Dai H., Liang Y., Parys C., Fernandez C., Wang M., Loor J.J. (2020). Supply of methionine and arginine alters phosphorylation of mechanistic target of rapamycin (mTOR), circadian clock proteins, and α-s1-casein abundance in bovine mammary epithelial cells. Food Funct..

[109] Long Y., Tsai W.-B., Chang J.T., Estecio M., Wangpaichitr M., Savaraj N., Feun L.G., Chen H.H.W., Kuo M.T. (2016). Cisplatin-induced synthetic lethality to arginine-starvation therapy by transcriptional suppression of ASS1 is regulated by DEC1, HIF-1α, and c-Myc transcription network and is independent of ASS1 promoter DNA methylation. Oncotarget.

[110] Mun G.I., Kim I.-S., Lee B.-H., Boo Y.C. (2011). Endothelial argininosuccinate synthetase 1 regulates nitric oxide production and monocyte adhesion under static and laminar shear stress conditions. J. Biol. Chem..

[111] Zhu Y., Zhou Z., Du X., Lin X., Liang Z.-M., Chen S., Sun Y., Wang Y., Na Z., Wu Z. (2025). Cancer cell-derived arginine fuels polyamine biosynthesis in tumor-associated macrophages to promote immune evasion. Cancer Cell.

[112] Chang K.-Y., Chiang N.J., Wu S.Y., Yen C.J., Chen S.H., Yeh Y.M., Li C.F., Feng X.X., Wu K., Johnston A. (2021). Phase 1b study of pegylated arginine deiminase (ADI-PEG 20) plus Pembrolizumab in advanced solid cancers. Oncoimmunology.

[113] Zhang Y., Chung S.-F., Tam S.-Y., Leung Y.-C., Guan X. (2021). Arginine deprivation as a strategy for cancer therapy: An insight into drug design and drug combination. Cancer Lett..

[114] Suo X., Lu X., Hu T., Ma G., Su Z. (2009). A solid-phase adsorption method for PEGylation of human serum albumin and staphylokinase: Preparation, purification and biochemical characterization. Biotechnol. Lett..

[115] Ni Y., Schwaneberg U., Sun Z.-H. (2008). Arginine deiminase, a potential anti-tumor drug. Cancer Lett..

[116] Hulin J.-A., Gubareva E.A., Jarzebska N., Rodionov R.N., Mangoni A.A., Tommasi S. (2019). Inhibition of Dimethylarginine Dimethylaminohydrolase (DDAH) Enzymes as an Emerging Therapeutic Strategy to Target Angiogenesis and Vasculogenic Mimicry in Cancer. Front Oncol..

